# Azathioprine as an adjuvant therapy in severe Graves’ disease: a randomized controlled open-label clinical trial

**DOI:** 10.3389/fendo.2023.1168936

**Published:** 2023-06-20

**Authors:** Magdy Mohamed Allam, Hanaa Tarek El-Zawawy, Amr Abdel Kader Okda, Ayoub Ali Alshaikh, Ramy Mohamed Ghazy

**Affiliations:** ^1^ Endocrinology Unit , Department of Internal Medicine, Alexandria University Student Hospital, Alexandria University, Alexandria, Egypt; ^2^ Endocrinology Unit, Department of Internal Medicine, Faculty of Medicine, Alexandria University, Alexandria, Egypt; ^3^ Department of Clinical Pharmacology, Faculty of Medicine, Alexandria University, Alexandria, Egypt; ^4^ Family & Community Medicine Department, King Khalid University, Abha, Saudi Arabia; ^5^ Tropical Health Department, High Institute of Public Health Alexandria University, Alexandria, Egypt

**Keywords:** antithyroid drugs, Graves’ disease, azathioprine, remission rate, relapse rate

## Abstract

**Introduction:**

Azathioprine (AZA) interferes with the activation of T and B lymphocytes, which are the main cells involved in the pathogenesis of Graves’ disease (GD). The aim of this study was to investigate the effectiveness of AZA as an adjuvant therapy to antithyroid drugs (ATDs) for moderate and severe GD. In addition, we conducted an incremental cost-effectiveness analysis of AZA to determine its cost-effectiveness.

**Methods:**

We conducted a randomized, open-label, and parallel-group clinical trial. We randomized untreated hyperthyroid patients with severe GD into three groups. All patients received 45-mg carbimazole (CM) as the starting dose and propranolol 40–120 mg daily. The first group (AZA1) received an additional 1 mg/kg/day AZA, the second group (AZA2) received an additional 2 mg/kg/day AZA, and the third group (control group) received only CM and propranolol. We measured thyroid-stimulating hormone (TSH) and TSH-receptor antibody (TRAb) levels at baseline and every 3 months, while free triiodothyronine (FT3) and free thyroxine (FT4) levels were measured at the time of diagnosis, 1 month after initiation of therapy, and every 3 months thereafter until 2 years after remission. Thyroid volume (TV) was assessed by ultrasound at baseline and 1 year after remission.

**Results:**

A total of 270 patients were included in this trial. By the end of follow-up, there was higher remission rate in the AZA1 and AZA2 groups compared with controls (87.5% and 87.5% *vs*. 33.4%, *p* = 0.002). Throughout the course of follow-up, FT3, FT4, TSH, and TRAb were significantly different between the AZA groups and the control group, but there was no significant difference regarding TV. The decline in the concentrations of FT4, FT3, and TRAb was significantly faster in the AZA2 group than in the AZA1 group. The relapse rate during the 12-month follow-up was insignificantly higher in the control group than in either the AZA1 or AZA2 group (10, 4.4, and 4.4%, *p* = 0.05, respectively). The median relapse time was 18 months for the control group and 24 months for the AZA1 and AZA2 groups. The incremental cost-effectiveness ratio for the AZA group compared with the conventional group was 27,220.4 Egyptian pounds per remission reduction for patients using AZA as an adjuvant for ATDs.

**Conclusion:**

AZA could be a novel, affordable, cost-effective, and safe drug offering hope for patients with GD to achieve early and long-lasting medical remission.

**Trial registry:**

The trial is registered at the Pan African Clinical Trial Registry (Registration number: PACTR201912487382180).

## Highlights

• Azathioprine is an adjuvant treatment to the standard antithyroid drug therapy that is a cost-effective and safe approach for patients with Graves’ disease to achieve early and long-lasting medical remission.

## Introduction

Graves’ disease (GD) is the most common cause of hyperthyroidism and approximately 3% of women and 0.5% of men develop GD in their lifetime (1, 2). GD is an autoimmune thyroid disorder where B and T lymphocyte–mediated autoimmunities are known to be coordinated at three well-known thyroid antigens: thyroglobulin, thyroid peroxidase, and the thyrotropin (TSH) receptor. The main pathogenic factor is the formation of autoantibodies which stimulate the TSH receptor on thyroid follicular cells, which are the cells responsible for producing thyroid hormones. In addition, anti-TSH receptor antibodies (TRAb) play a role in promoting Graves’ ophthalmopathy (GO) by stimulating the TSH receptor on thyroid follicular cells (1, 2).

Antithyroid drugs (ATDs) are essential in the treatment of GD. They suppress the production of thyroid hormones by inhibiting the enzymes involved in their synthesis, such as thyroxine and triiodothyronine. Presently, treatment strategies primarily rely on ATDs or radioactive iodine (RAI) therapy to reduce thyroid hormone production. However, there is a need for greater attention to be paid to the underlying immunological mechanisms involved in GD, such as the role of T and B cells in autoimmunity (3). ATDs are the least invasive treatment option and are usually the first-line treatment for duration of 12–18 months. Despite their effectiveness, there is a high risk of relapse (around 50%) even after this duration of treatment with ATDs. Patients may also experience medication side effects, especially when treated with higher doses, such as hepatic injury and agranulocytosis (4).

The TRAb level is a useful predictor for remission and relapse of patients at the end of the course of ATDs therapy. Patients with persistent elevation of TRAb level are unlikely to achieve remission, with relapse rates approaching 80–100%. On the other hand, patients with low or undetectable TRAb have a higher probability of achieving permanent remission, with relapse rates approaching 20–30% (5). In cases where patients have persistently high TRAb levels, they could continue ATD therapy and repeat TRAb testing after additional 12–18 months. Alternatively, they may choose to undergo an alternate definitive therapy such as RAI or surgery to achieve permanent remission (6).

The high relapse rate after ATD withdrawal implies that patients may need to resume ATD or resort to definitive treatment such as RAI or total thyroidectomy. Successful thyroid ablation by RAI or total thyroidectomy necessitates lifelong thyroid hormone replacement therapy. However, a small percentage of levothyroxine-treated patients (about 5–10%) experience persistent hypothyroid symptoms despite normal serum TSH levels (7). RAI therapy is associated with an increased risk of progression to or *de novo* development of GO, especially in at-risk patients (8). Low-dose steroids could be used as prophylaxis for RAI-associated GO progression (9). The current treatment options for GD do not target the key immunopathogenic mechanisms. Recently, some biological agents such as the monoclonal antibody K1-70 and the peptide ATX-GD-59 have been developed to treat GD (10).

Azathioprine (AZA) is a synthetic purine analog with immunosuppressive properties that is commonly used to treat autoimmune diseases such as rheumatoid arthritis, inflammatory bowel disease, multiple sclerosis, myasthenia gravis, and certain cancers. AZA interferes with the metabolism of purine nucleic acid, which is essential for lymphoid cell proliferation following antigenic stimulation, resulting in the inhibition of cellular immunity and antibody responses (10). Based on these pharmacological properties, AZA has been studied as a potential adjuvant therapy for GD, in addition to its known benefits in the management of GO, as demonstrated in previous studies (11, 12). AZA has a generally good safety and tolerability profile, along with therapeutic and cost advantages over other immunosuppressants. The most common side effects of AZA are gastrointestinal disturbances, which can be minimized by adjusting the dosage or taking the medication with food (13). Seven trials comprising 862 patients were included in a meta-analysis to assess the role of immunosuppressive in management of GD. The data were pooled using a random-effects model, and the analysis revealed a risk ratio of 0.55 (95% CI 0.41–0.75) for recurrence rate of GD in patients receiving immunosuppressive drugs. Only one of the trials used AZA in the treatment of GD, but it had a small sample size, a moderate to high risk of bias, and was mostly heterogeneous and non-randomized. Moreover, adverse effects were not systematically reported in this trial. Nonetheless, the results remained consistent in subgroup analyses for trials using both corticosteroids and non-corticosteroid drugs, as well as for randomized and non-randomized controlled trials (14). These findings are promising and suggest that the addition of immunosuppressive drugs to the ATDs regimen may be beneficial for GD treatment. However, before such protocols are used in standard care, large-scale validation in a high-quality randomized trial is needed. Furthermore, there are some case reports that suggest the potential usefulness of AZA in severe cases of Graves’ hyperthyroidism (15, 16). This study aimed to assess the efficacy of addition of AZA to the conventional ATDs in moderate and severe Graves’ hyperthyroidism.

## Methods

### Study design and participants

This is a randomized, open-label, and parallel-group clinical trial. We followed the CONSORT checklist of reporting of randomized control trial (17). The patients included in this study were recruited from two endocrine outpatient clinics, namely, the Alexandria University Students Hospital (AUSH) and Alexandria Main University Hospital (AMUH) during the period between January 2017 and December 2019. Inclusion criteria: We included moderate and severe GD patients defined as those who exhibited extreme symptoms (such as heart rate > 120 beats/min, hyperthyroid heart disease, or weight loss > 10 kg) and/or had free T4 or free T3 levels that exceeded two times the upper limit of normal (i.e., FT4 ≥ 3.6 ng/dl or FT3 ≥ 8.4 pg/ml, respectively). Additionally, married women in their childbearing period were required to have a negative pregnancy test during the first screening visit and to use a highly effective method of contraception throughout the entire study period and for at least 90 days after the last dose of AZA.

We excluded pregnant, breastfeeding, attempting to conceive, had end-stage diseases, had an active or chronic infectious disease, had any disease that compromises immune function, and had hemoglobin concentration below 12 g/dl, platelet count below 130 × 10^9^/liter, white cell count below the local laboratory’s reference range, and lymphocyte count below 0.8 × 10^9^/liter. Patients who had recently received or planned to receive live vaccines, AZA, cytokine or anti-cytokine therapy, or corticosteroids (including for severe orbitopathy) within the three months prior to the study were also excluded, as well as patients with typical symptoms and signs of thyroid storm.

### Sample size and randomization

The study used G power software version 3.1.9.4 and was based on previous research that found a relapse rate of 23.5% for patients receiving immunosuppressive drugs compared with 59.1% for control patients with GD. With an alpha error of 0.05, power of 95%, and response rate of 30%, the minimum required sample size for each group was determined to be 68. A computer program generated a list and randomly allocated patients into three groups matched for age, sex, and TRAb. The first group (*AZA1*) received 45-mg carbimazole (CM) + propranolol 40–120 mg + 1 mg/kg/day AZA. The second group (*AZA2*) received 45 mg CM + propranolol 40–120 mg + 2 mg/kg/day AZA. The third group (*control group*) received 45 mg CM + propranolol 40–120 mg.

### Study procedure

We collected sociodemographic data from the study participants such as age, sex, and smoking history (mild is less than 10 cigarettes per day and heavy is equal or more than 20 cigarettes per day). In addition, we evaluated patients for GO based on the European Group on Graves’ orbitopathy (EUGOGO) clinical practice guidelines (18). All patients in the study received an initial dosage of 45 mg/day of CM and propranolol 40–120 mg/day. TSH and TRAb levels were measured at the time of diagnosis and every 3 months during the study. FT3 and FT4 levels were also measured at the time of diagnosis, 1 month after the start of therapy, and every 3 months up to 2 years after remission. We measured serum thyroid profile using an enzyme-linked immunosorbent assay (ELISA), (Monobind Inc. California). Their reference ranges were as follow: TSH: 0.39-4.16 mIU/liter, FT3: 1.4–4.2 pg/ml, FT4: 0.8–1.8 ng/dl, and TRAb: < 1.75 U/liter. The dosage of CM was adjusted based on the FT3 and FT4 levels by 5–10 mg/day to maintain euthyroidism and gradually tapered until it was stopped when TRAb, FT3, FT4, and TSH levels remained within the reference range for at least 6 months at the minimum maintenance dosage. The thyroid volume (TV) was evaluated by the same radiologist and ultrasound device at baseline and 1 year after remission. AZA was discontinued 6 months after CM withdrawal, and treatment with CM was continued for a maximum of 12 months. After stopping CM, serum FT4 and TSH levels were measured once every 4 months during the following 2 years to monitor the patients’ euthyroid status. Patients who remained euthyroid for 1 year were considered to have achieved remission. The patients were followed up for 2 years after remission to ensure the maintenance of remission and to detect any relapse.

To ensure standardization of the ATDs, we chose CM as the preferred treatment based on the American Thyroid Association (ATA) guidelines, which recommend it as the preferred ATDs for adults due to its reduced risk of major side effects compared with propyl thiouracil (PTU) (19). Compliance with the medication regimen was monitored by directly questioning the patient and a family member at each clinic visit and by inspecting their pill bottles.

### Study outcomes

*The primary outcome* of the trial was to determine the rates of partial and complete remission of GD within 12 months. *Complete remission* was defined as being in a state of normal thyroid function with negative TRAb for at least 1 year after stopping CM. *Partial remission* was defined as being in a state of normal thyroid function while receiving a low dose of CM (5–15 mg/day) for at least 12 months, even with positive TRAb. *Treatment failure* was defined as being in a hyperthyroid state or a normal thyroid state while receiving more than 15 mg CM (5–10 mg/day) for at least 12 months, even with positive TRAb. *Relapse* was defined as the recurrence of hyperthyroidism at any time following remission (19).

*The secondary outcomes* of the trial were to determine the duration of treatment needed to normalize serum thyroid hormones (TSH, FT3, and FT4) to their reference ranges, assess the rate of relapse, and evaluate the cost-effectiveness of adding AZA to the treatment regimen. Safety and tolerability of the combination therapy were also considered as secondary outcomes. Adverse events (AEs) were coded using the International Conference on Harmonization (ICH) Guidelines. AEs and were monitored at every visit until resolution or until the patient was lost to follow-up. We prioritized patient safety while using AZA as an adjuvant therapy for GD by conducting regular monitoring for potential side effects. This included monthly evaluations of peripheral blood counts, kidney function, and liver function through blood tests. AEs were classified into persisting pruritus and rashes despite antihistaminic treatment, hepatic dysfunction (total bilirubin > 3.0 mg/dl, or > threefold elevation of aspartate aminotransferase (AST) and/or alanine aminotransferase (ALT) over the reference range), and granulocytopenia or agranulocytosis (defined as a neutrophil count of 1000/mm^3^ or lower for granulocytopenia and 500/mm^3^ or lower for agranulocytosis). Patients who withdrew from the study due to AE or worsening hyperthyroidism were included in the primary analysis (19). The outcome assessor was blind; he was kept unaware of the treatment allocation or group assignment of the participants.

### Ethics

The study follows the principles of the Declaration of Helsinki and its later amends. The study protocol was approved by the local ethics committee at Alexandria University Faculty of Medicine and Alexandria University High Institute of Public Health. The trial is registered at the Pan African Clinical Trial Registry (registration number: PACTR201912487382180). After an explanation of the treatment method and AEs of AZA and CM, written consent was obtained from all patients.

### Statistical analysis

The primary analysis was based on the intention-to-treat allocation. The distribution of metric variables was characterized by reporting means and standard deviation or median and quartiles as appropriate. For dichotomous variables, absolute and relative frequencies were presented. The Mann–Whitney *U* test is a non-parametric test was used to compare two groups when the data were not normally distributed. The two-sided chi-square test was used to compare the frequencies of two categorical variables. Fisher’s exact test is a statistical significance test was used to determine the association between two categorical variables when the assumptions of chi-square test were violated. Remission proportions, relapse rate, the change of TV, and AEs incidences among responders were compared by Fisher’s exact test and OR with 95% CI. All reported *p*-values were two-sided. The significance level was set at 0.05. Statistical analysis was performed with the SPSS/PC software package for MS Windows (IBM Corp. released 2013, IBM SPSS Statistics for Windows, version 22.0).

Kaplan–Meier curves were used to analyze time-to-event data, such as the time-to-remission. In this study, the curves plot the cumulative proportion of individuals who experience remission of GD over time. Log-rank testing was the statistical test used to compare the survival curves of the studied groups. Significant *p*-value suggests that the time-to-event differs between the groups.

Economic analysis: An incremental cost-effectiveness analysis (ICER) was conducted to evaluate the costs and effectiveness of AZA. The analysis was performed from the perspective of the health sector, using Egyptian pounds (EGP) as the currency. The analysis considered all direct and indirect costs. Direct costs of AZA included medication costs, definitive treatment (including no medicine, surgery which cost 30000 EGP, or RAI which cost 8000 EGP), replacement treatment, investigations (which cost 565 EGP), and clinic visits (which cost 250 EGP). Indirect costs included the number of absenteeism days before returning to normal activities and work (multiplied by 100 EGP). The effectiveness of AZA was measured by the rate of remission. The total costs for each of the AZA1 and conventional groups were calculated by summing each individual patient’s cost. The ICER was calculated using the formula: ICER = (CA – Cc)/(EA – Ec), where CA is the cost of AZA until definitive treatment, Cc is the cost of conventional therapy until definitive treatment, EA is the rate of remission for AZA, and Ec is the rate of remission for conventional therapy.

## Results

### Clinical characteristics of the study subjects

**Figure 1:**
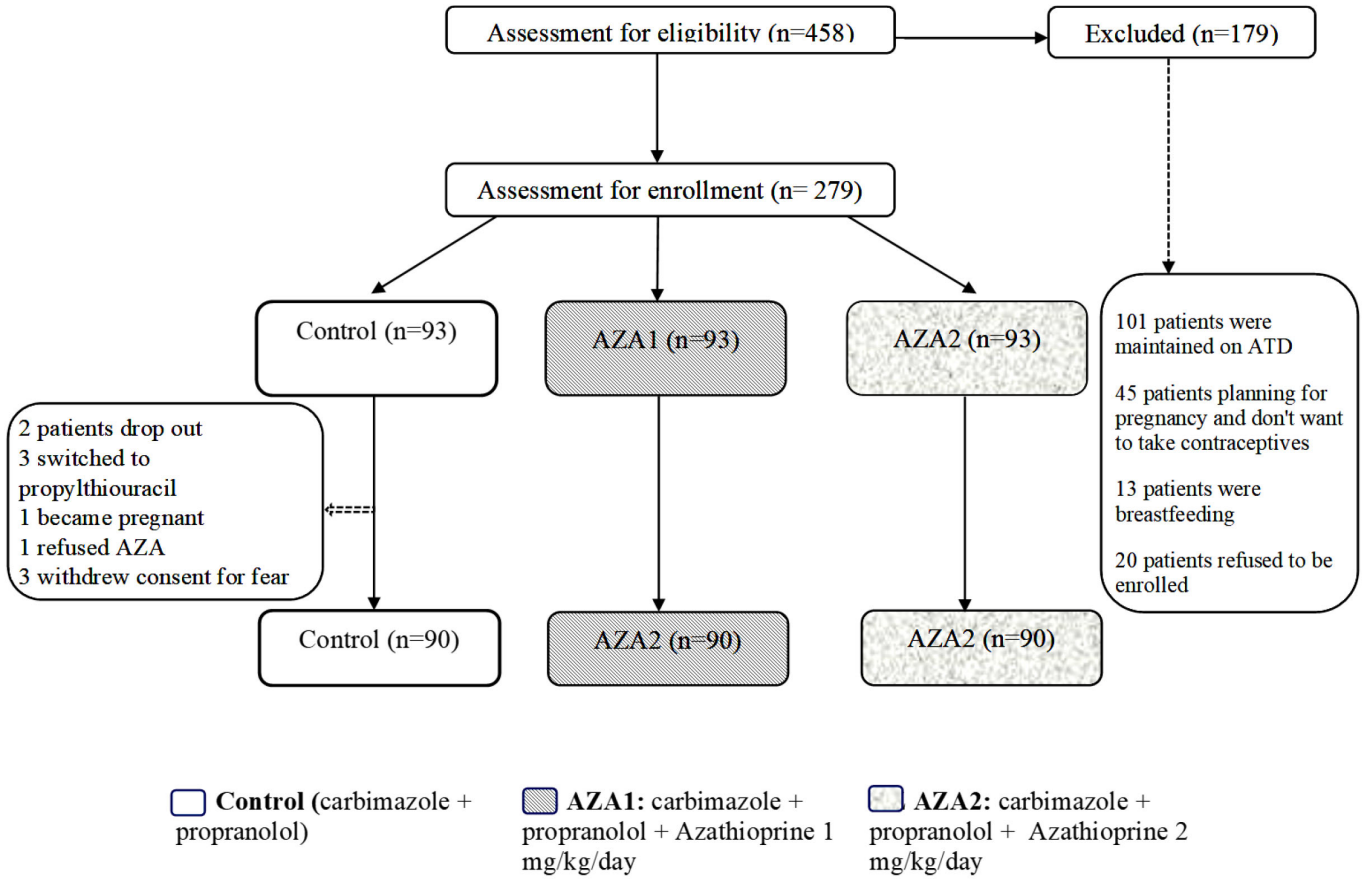
Flow chart of the study periods and participants.

In total, 458 patients were screened, and 279 of them met our inclusion criteria and were enrolled in the study. Out of these, 270 subjects completed the study, while nine patients dropped out (two were lost to follow-up, three switched to propylthiouracil due to their desire for pregnancy, one female refused to continue taking contraceptives, and three withdrew consent due to fear of side effects). These nine patients were considered as non-responders and included in the intention-to-treat analysis. **Figure 1** The baseline characteristics of all three groups were broadly similar (**Table 1**).

**Table 1:** Comparison between the studied groups regarding demographic data, thyroid profile, and Graves’ ophthalmopathy.

		ATDs only (control group) (*N* = 90)	ATDs + Azathioprine (1 mg/dl) (*N* = 90)	ATDs + Azathioprine (2 mg/dl) (*N* = 90)
		Mean ± SD	Mean ± SD	Mean ± SD
Age (years)		45.3 ± 13.9	43.6 ± 13.3	41.1 ± 12.2
SBP (mmHg)		125.3 ± 28.3	118.6 ± 15.7	112.9 ± 22.2
DBP (mmHg)		78.0 ± 10.8	75.7 ± 9.8	74.3 ± 7.9
Pulse (beat per minute)		115 ± 10.3	118.0 ± 11.9	130.0 ± 21.4
FT4 (ng/ml)		4.98 ± 4.2	3.9 ± 2.1	5.9 ± 3.7
FT3 (pg/ml)		9.4 ± 5.9	7.5 ± 4.0	11.2 ± 5.0
TSH (mU/liter)*10^3^		0.004 ± 0.001	0.008 ± 0.002	0.001 ± 0.002
TRAb (IU/liter)		31.4 ± 19.5	25.6 ± 25.7	36.5 ± 20.2
Thyroid volume		69.4 ± 35.4	51.6 ± 20.2	79.7 ± 47.2
WBCs (×10^9^/liter)		5.2 ± 1.4	7.7 ± 2.6	6.5 ± 4.2
ALT (U/liter)		11.2 ± 5.1	10.6 ± 7.7	10.8 ± 5.9
AST (U/liter)		10.7 ± 6.8	12.1 ± 5.3	11.7 ± 4.1
		Percent (*N*)	Percent (*N*)	Percent (*N*)
Females		65.6 (59)	70.0 (63)	55.6 (50)
Smoking	Non-smoker	90.0 (81)	86.7 (78)	75.6 (68)
	Mild smoking	3.33 (3)	1.1 (1)	12.3 (11)
	Heavy smoking	6.7 (6)	12.2 (11)	12.2(11)
Grave`s ophthalmopathy		12.2 (11)	6.7 (6)	25.6(23)

N, Number of patients; ATD, Antithyroid drugs; SBP, Systolic blood pressure; DBP, Diastolic blood pressure; FT4, free T4; FT3, free T3; TSH, thyroid stimulating hormone; WBCs, white blood cells; ALT, alanine transaminase.; AST, Aspartate transaminase.

### Primary outcome

**Figure 2:**
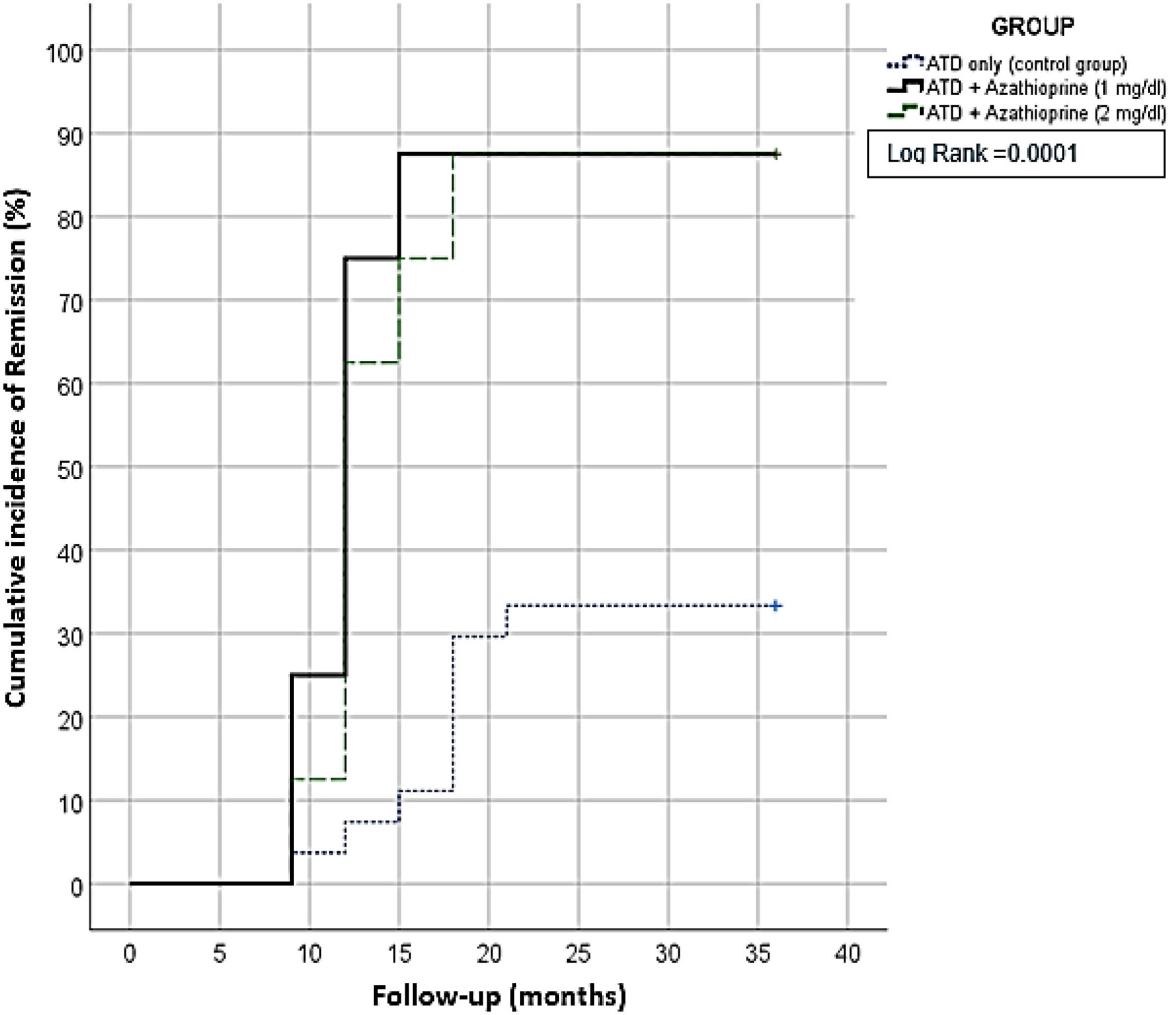
Kaplan-Meier survival curve for three years; showed patients receiving Azhatioprine add on had a significant higher remission rate.

During the follow-up period, the difference in the binary clinical composite outcome was measured to compare the remission rate. Results showed that 50% of patients in the AZA groups achieved remission within the first year of treatment, while only 5% of the control group showed remission. By the end of the follow-up period, a higher remission rate was observed in the AZA1 and AZA2 groups compared with the control group (87.5%, 87.5% *vs*. 33.4%, *p* = 0.002), as presented in **Table 2** and **Figure 2**. The number needed to treat with longer term AZA + CM was 1.2 per remission.

**Table 2:** Comparison between the three studied groups for 2 years after remission.

Fate % (*N*)	ATD only (control group) (*N* = 90)	ATD + Azathioprine (1 mg/dl) (*N* = 90)	ATD + Azathioprine (2 mg/dl) (*N* = 90)	*p*
**Complete remission**	7.4(7)	87.8(79)	68.9(62)	0.001
**Partial remission**	25.9(23)	0.0(0)	24.4(22)	
**Failure**	66.7(60)	12.2(11)	6.7(6)	
**Relapse rate**	10.0 (9)	4.4(4)	4.4 (4)	0.0508
**Median relapse time (month)[IQR]**	18 (12–30)	24 (18–36)	24 (24–36)	

IQR, Interquartile range.

### Secondary outcomes

**Figure 3:**
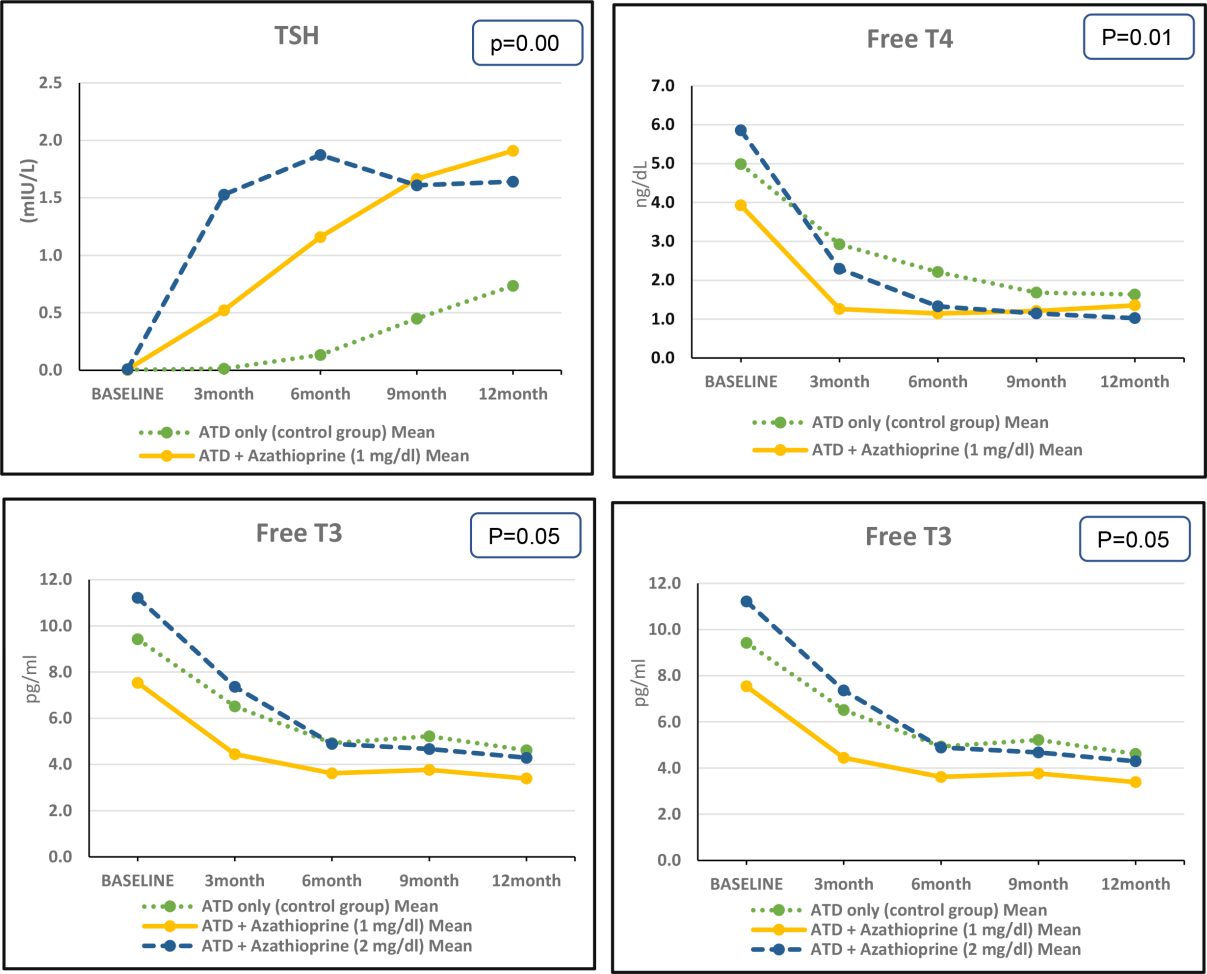
Changes in thyroid parameters in the three studied groups along 1 year.

Throughout the follow-up period, there were significant differences in the concentrations of FT3, FT4, TSH, and TRAb between the AZA1, AZA2, and control groups. However, there were no significant differences in TV between the groups. At the end of the follow-up, there were no significant differences in the concentrations of FT3, FT4, and TSH between the AZA1 and AZA2 groups. However, the concentration of TRAb was significantly lower in the AZA1 group compared with the AZA2 group. **Figure 3** and **Supplementary Table S1**.

We found that after 3 months of therapy, the decrease in FT4 concentration was significantly faster in the AZA2 group compared with the AZA1 group, with a mean difference of 3.56 ng/dl versus 2.67 ng/dl, respectively (*p* = 0.034). Similarly, the decrease in FT3 concentration was significantly faster in the AZA2 group compared with the AZA1 group, with a mean difference of 3.85 pg/ml versus 3.09 pg/ml, respectively (*p* = 0.001). Similar pattern of improvement was observed in TSH, the increase in TSH concentration was significantly faster in the AZA2 group compared with the AZA1 group, with a mean difference of 1.519 mU/liter versus 0.514 mU/liter, respectively (*p* = 0.07). Moreover, the decrease in TRAb concentration was significantly faster in the AZA2 group compared with the AZA1 group, with a mean difference of 15.85 U/liter versus 15.08 U/liter, respectively (*p* = 0.001). Based on the thyroid ultrasound results, there was no significant difference in TV between the study groups at baseline or by the end of the 12-month follow-up period. **Supplementary Table S1**.

At the end of the 12-month follow-up period, the relapse rate was slightly higher in the control group compared with either the AZA1 or AZA2 group, but the difference was not statistically significant (10.0%, 4.4%, 4.4%, *p* = 0.05, respectively). The median relapse time was 18.0 (12.0–30.0) months for the control group, while it was 24.0 (18.0–36.0) months for both AZA1 and AZA2 groups. **Table 2**. The frequency of side effects such as gastrointestinal disturbances, leucopenia, and infection did not show any significant difference among the groups studied, *p* > 0.05. **Table 3**.

**Table 3:** Adverse effects of the medications used in the study.

Adverse effects	ATDs only (control group) (*N* = 90)	ATDs + Azathioprine (1 mg/dl) (*N* = 90)	ATDs + Azathioprine (2 mg/dl) (*N* = 90)	*p*
	Percent (*n*)			
Gastrointestinal disturbance	4.4 (4)	4.44(4)	5.6 (5)	0.608
Leucopenia	7.8(7)	2.2(2)	6.7 (6)	
Infections	14.4(13)	8.9 (8)	12.2(11)	

**Figure 4:**
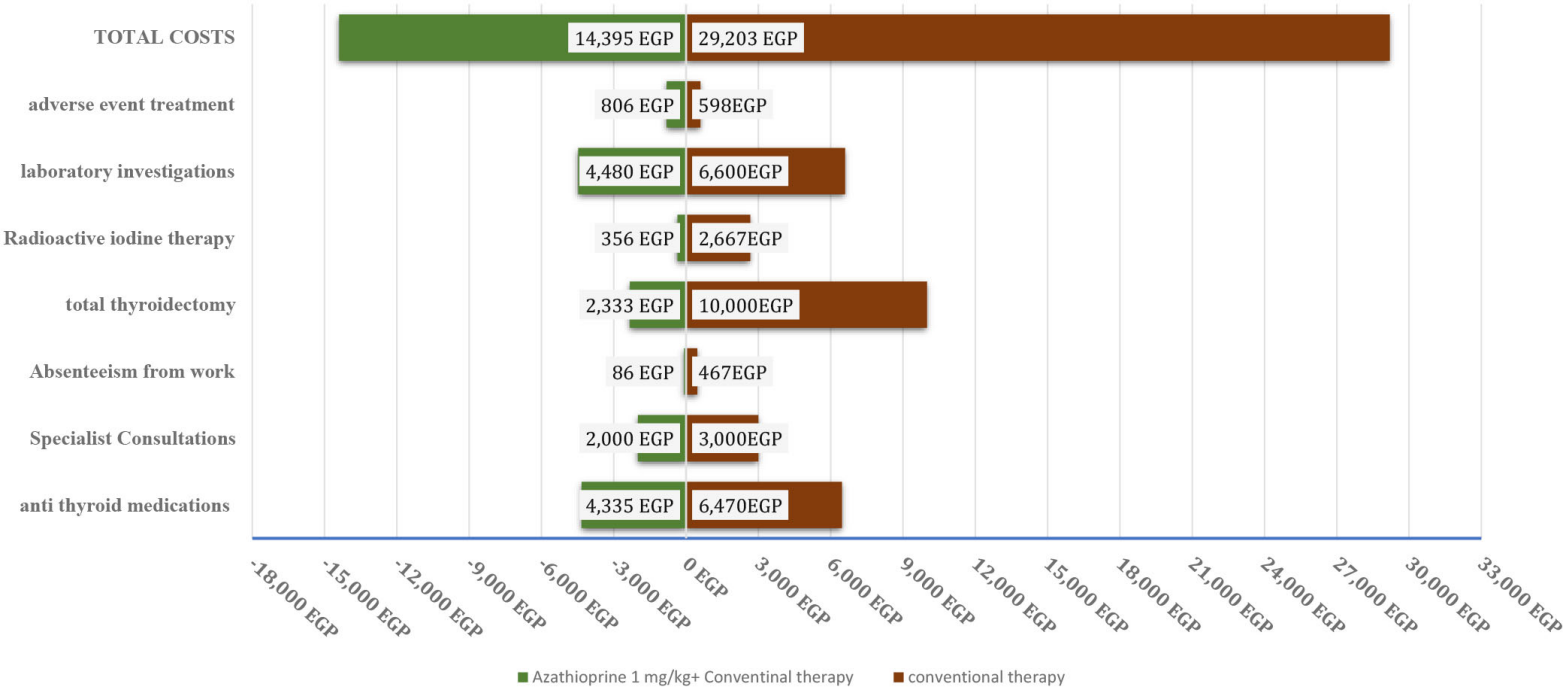
A tornado diagram of different costs on exposure to Azhatioprine and conventional therapy versus conventional therapy only for three years of follow up. EGP, Egyptian pound.

Adding AZA to conventional therapy resulted in a significant decrease in the average cost of Graves’ disease treatment compared with conventional therapy alone. This decrease was due to a lower frequency of specialist consultations, lower costs of definitive treatment (such as thyroidectomy and RAI), and fewer absenteeism days (*p* < 0.010, *p* < 0.010, and *p* < 0.030, respectively) per month. Adding adjuvant AZA to conventional treatment significantly increased the remission rate, which in turn led to a reduction in total costs. The average patient cost for the AZA group was 14,395.45 EGP (equivalent to 1,744.9 USD), compared with 29,203.34 EGP (equivalent to 3,539.8 USD) for the conventional treatment group. The ICER for the AZA group compared with the conventional group was 27,220.4 EGP/remission (equivalent to 3,299.39 USD/remission), indicating that using AZA as an adjuvant for ATDs is a cost-effective option for patients. **Figure 4**.

## Discussion

Although ATDs, surgical thyroidectomy, and RAI therapy are the traditional methods widely used for the treatment of GD, they have lifelong impacts on patients. ATDs are associated with side effects and a high rate of GD recurrence, while RAI ablation and thyroidectomy can result in permanent hypothyroidism. In recent years, new agents such as biologics, small-molecule peptides, and immunomodulators have emerged as potential treatments for GD. However, these new treatments also have their own limitations (20).

Our study introduces a promising therapeutic approach for the management of GD by adding AZA as an adjuvant to ATDs treatment. We found that this approach led to significantly higher rates of remission compared with conventional treatment alone. Additionally, the relapse rate in patients who received AZA as an adjuvant therapy was lower than that of the control group, although this difference was not statistically significant. Additionally, adjuvant AZA therapy is associated with a reduction in the total cost of treatment compared with conventional therapy alone.

AZA has been used for the treatment of GO for a long time. Recent studies have investigated the mechanisms of AZA and found that it can induce apoptosis of CD4+ T lymphocytes by modulating the activation of Rac1 (Ras-related C3 botulinum toxins substrate 1) (3). In a review by Krause et al. (21), they demonstrated that TRAb was an important modulator of GD and concluded that blocking TRAb can be a promising combined therapeutic approach with ATDs to achieve remission in GD. The study by Lane et al. (22) highlighted the potential of new therapeutic options, such as biologics, small molecules, and peptide immunomodulation, which target TRAb, as a promising approach for the treatment of GD.

Our study found that patients with GD who received AZA in combination with ATDs had significantly lower levels of TRAb compared with those who only received ATDs (87.5, 87.5, and 33.4%), respectively. The remission rate after ATDs was only 33.4% which is lower what was reported in literature. The rate of remission of Graves’ hyperthyroidism after a course of ATDs is less than 50% in clinical trials (23) and roughly 30% in clinical settings (24). This variation can be attributed to several factors, such as different definitions of remission used across these studies, variations in the methods used to test TRAb, and differences in the duration of follow-up. Franklyn and Boelaert 2012 (4) defined remission as follow normal thyroid function tests 1 year after ATDs withdrawal, while Sjölin et al. (25) defined remission based on achieving euthyroidism within 3–6 months after intervention, which included the last ATDs, surgery, or RAI. In addition, patients who did not reappear in the files and did not report relapse in their questionnaire were considered to be in remission. Another explanation is that many factors may affect the remission such as severe biochemical disease, male sex, young age (< 40 years) (24), high concentrations of TSHR antibodies, presence of large goiter, and smoking (26, 27).

This finding suggests that the use of AZA may contribute to higher remission rates, as TRAb is considered to be the most important predictor of remission in GD. The study conducted by Cappelli et al. (28) found that the rate at which TRAb levels decrease was the most accurate predictor of remission in GD, with high sensitivity and specificity. Also, other studies showed that TRAb level was a prognostic marker of attaining remission in GD (29, 30). Our finding highlighted the potential of AZA as a cheaper valuable option for the management of GD, particularly in cases where conventional therapy has failed or was associated with a high risk of adverse effects. Furthermore, the study shaded the light on the potential mechanism of action of AZA in modulating TRAb levels, which could help pave the way for the development of new therapeutic options for GD.

A review by Barbesino and colleagues (31) discussed a randomized controlled study that demonstrated the beneficial effect of long-term AZA use in patients with thyroid eye disease who continued the drug for more than 24 weeks. This finding suggests that AZA has a long-term effect, which is consistent with the results of our study. Additionally, Struja et al. (32) conducted a systematic review and meta-analysis, which supports our study findings. They found that the addition of immunosuppressive drugs to standard GD treatment can significantly reduce the risk of relapse in GD patients. This study highlighted the potential benefits of using AZA as an adjuvant therapy in managing GD, consistent with another research. Our finding demonstrated the added advantage of AZA being more affordable and accessible drug.

Despite the achieved remission with AZA, the reduction of thyroid gland volume was not statistically significant. In the same vein, Benker et al. (33) examined the predictors of remission in Graves’ hyperthyroidism, baseline factors were evaluated to determine their association with subsequent remission (34). The study included 313 patients who were followed for an average of 4.3 years, with a relapse rate of 58% (similar in both the 10 mg/day and 40 mg/day groups). The study found no significant differences at baseline between patients who eventually experienced relapse and those who achieved sustained remission in term of TV. On the other hand, Sakane et al. (34), reported a significant correlation between TV and both serum thyroglobulin concentration TRAb activity. Most patients experienced a gradual decrease in TV during ATDs therapy. Of the 20 patients who received treatment, 11 achieved remissions after discontinuing therapy, and their TV was significantly smaller (*p* < 0.01) than before treatment.

Despite being an effective adjuvant therapy for GD, AZA can cause side effects in some patients. Nausea is the most common side effect, followed by fever, fatigue, arthralgia, leucopoenia, rash, infection, hepatotoxicity, and renal damage, which can affect 15–28% of patients (35). Therefore, regular monitoring of patients receiving AZA is crucial to detect and manage any potential complications promptly. In this study, the incidence of side effects was not statistically significant between the intervention and control groups. This finding highlights the potential of AZA as a new modality to treat GD, as it demonstrates that the benefits of AZA outweigh the risks of developing side effects.

### Strengths and limitations

While there are several studies and reviews in the literature that have demonstrated the role of AZA in the management of GO, ours is the first multi-center study to investigate the effect of AZA, at specific doses, on the remission of GD. Another point of strengths was the large number of patients included, as well as the clinical homogeneity of the sample. Homogeneity of the sample ensures that all patients had similar characteristics. It can also improve the internal validity of the study and reduce the risk of confounding factors. Additionally, the study design included randomization of the study participants. Randomization is a key feature of clinical trials that helps to minimize bias and ensure that the treatment groups are comparable. However, a limitation of the study is that the follow-up period was only 12 months after the cessation of treatment, which may have been relatively short to evaluate long-term remission status, even though most GD recurrences tend to occur within the first 12 months after ATD withdrawal (32, 36). Also as, there was no blinding of participants or researchers there might be an unintentional influence on the study outcomes.

## Conclusion

Adding AZA to ATD appears to be a promising approach to achieve rapid TSH control and an effective early reduction in TRAb levels, potentially resulting in early and long-lasting remission rates in patients with GD. This combined therapy could offer a novel approach that is cost-effective compared with other options such as thyroidectomy and RAI.

## Data availability statement

The original contributions presented in the study are included in the article/**Supplementary Material**. Further inquiries can be directed to the corresponding author.

## Ethics statement

The studies involving human participants were reviewed and approved by Alexandria University. The patients/participants provided their written informed consent to participate in this study.

## Author contributions

MA: Adopted the research idea, participated in the patient management and data collection, contributed to the interpretation of cases, and critically reviewed the manuscript. HE-Z: Participated in data collection, contributed to the interpretation of the cases, wrote and critically reviewed the manuscript. AO: has participated in developing the methodology, interpretation of the results, and critically reviewed the manuscript. RG: has participated in developing the methodology, interpretation of the results, and critically reviewed the manuscript. AA: has participated in interpretation of the results, and critically reviewed the manuscript. All authors contributed to the article and approved the submitted version.
