# Supplementary material for: Azathioprine as an adjuvant therapy in severe Graves’ disease: a randomized controlled open-label clinical trial
Source: Front Endocrinol (Lausanne). 2023 Jun 20;14:1168936. doi: 10.3389/fendo.2023.1168936 (PMC10319122; doi:10.3389/fendo.2023.1168936)
Supplement: Supplementary file 1 [file Table_1.docx]

**Table 1S: Comparison of thyroid parameters between the studied groups**

| TSH  (mU/L) | | ATD only (control group)  (N =90) | | ATD + Azathioprine (1 mg/dl)  (N =90) | | ATD + Azathioprine (2 mg/dl)  (N =90) | | Sig. | P1 | P2 | P3 |  |
| --- | --- | --- | --- | --- | --- | --- | --- | --- | --- | --- | --- | --- |
| GROUP | Mean | | SD | Mean | SD | Mean | SD |  |  |  |  |  |
| Baseline | 0.004 | | 0.001 | 0.008 | 0.002 | 0.0010 | 0.002 | 0.034 | 0.1319 | 0.0544 | 0.6023 |  |
| 3months | 0.014 | | 0.290 | 0.522 | 0.532 | 1.529 | 0.532 | 0.053 | 0.4068 | 0.0166 | 0.1885 |  |
| 6monthss | 0.135 | | 0.382 | 1.159 | 0.703 | 1.873 | 0.703 | 0.081 | 0.2079 | 0.0358 | 0.4765 |  |
| 9months | 0.449 | | 0.214 | 1.664 | 0.393 | 1.609 | 0.393 | 0.005 | 0.0081 | 0.0119 | 0.9040 |  |
| 12months | 0.736 | | 0.191 | 1.910 | 0.350 | 1.641 | 0.350 | 0.009 | 0.006 | 0.045 | 0.499 |  |
| P1==> ATD only (control group) VS CONTROL VS ATD + Azathioprine (1 mg/dl) P2==> ATD only (control group) VS CONTROL VS ATD + Azathioprine (2 mg/dl) P3==> CONTROL VS ATD + Azathioprine (1 mg/dl) VS ATD + Azathioprine (2 mg/dl) | | | | | | | | | | | |  |
|  |  |  |  |  |  |  |  |  |  |  |  |  |

| Free T4 | ATD only (control group) | | ATD + Azathioprine (1 mg/dl) | | ATD + Azathioprine (2 mg/dl) | | Sig. | P1 | P2 | P3 |  |
| --- | --- | --- | --- | --- | --- | --- | --- | --- | --- | --- | --- |
| GROUP | Mean | SD | Mean | SD | Mean | SD |  |  |  |  |  |
| Baseline | 4.983 | 4.240 | 3.926 | 2.124 | 5.854 | 3.693 | 0.608 | 0.064 | 0.631 | 0.260 |  |
| 3months | 2.927 | 1.374 | 1.260 | 0.715 | 2.295 | 1.943 | 0.018 | 0.005 | 0.271 | 0.149 |  |
| 6months | 2.211 | 1.304 | 1.145 | 0.235 | 1.326 | 0.677 | 0.025 | 0.020 | 0.051 | 0.742 |  |
| 9months | 1.681 | 0.684 | 1.212 | 0.401 | 1.143 | 0.304 | 0.034 | 0.055 | 0.029 | 0.817 |  |
| 12months | 1.634 | 0.758 | 1.354 | 0.292 | 1.024 | 0.266 | 0.060 | 0.279 | 0.021 | 0.302 |  |
| P1==> ATD only (control group) VS CONTROL VS ATD + Azathioprine (1 mg/dl) P2==> ATD only (control group) VS CONTROL VS ATD + Azathioprine (2 mg/dl) P3==> CONTROL VS ATD + Azathioprine (1 mg/dl) VS ATD + Azathioprine (2 mg/dl) | | | | | | | | | | |  |
|  |  |  |  |  |  |  |  |  |  |  |  |

| Free T3 | ATD only (control group) | | ATD + Azathioprine (1 mg/dl) | | ATD + Azathioprine (2 mg/dl) | | Sig. | P1 | P2 | P3 |  |
| --- | --- | --- | --- | --- | --- | --- | --- | --- | --- | --- | --- |
| GROUP | Mean | SD | Mean | SD | Mean | SD |  |  |  |  |  |
| Baseline | 9.427 | 5.861 | 7.541 | 4.006 | 11.219 | 4.989 | 0.408 | 0.394 | 0.417 | 0.183 |  |
| 3months | 6.517 | 2.822 | 4.445 | 1.781 | 7.365 | 4.580 | 0.147 | 0.101 | 0.496 | 0.064 |  |
| 6months | 4.929 | 2.283 | 3.619 | 1.125 | 4.893 | 3.236 | 0.372 | 0.171 | 0.969 | 0.281 |  |
| 9months | 5.220 | 2.318 | 3.765 | 1.580 | 4.675 | 2.164 | 0.257 | 0.105 | 0.538 | 0.409 |  |
| 12months | 4.614 | 1.790 | 3.393 | 1.085 | 4.294 | 1.746 | 0.209 | 0.079 | 0.639 | 0.290 |  |
| P1==> ATD only (control group) VS CONTROL VS ATD + Azathioprine (1 mg/dl) P2==> ATD only (control group) VS CONTROL VS ATD + Azathioprine (2 mg/dl) P3==> CONTROL VS ATD + Azathioprine (1 mg/dl) VS ATD + Azathioprine (2 mg/dl) | | | | | | | | | | |  |
|  |  |  |  |  |  |  |  |  |  |  |  |

| TSH receptor antibodies | ATD only (control group) | | ATD + Azathioprine (1 mg/dl) | | ATD + Azathioprine (2 mg/dl) | | Sig. | P1 | P2 | P3 |  |
| --- | --- | --- | --- | --- | --- | --- | --- | --- | --- | --- | --- |
| GROUP | Mean | SD | Mean | SD | Mean | SD |  |  |  |  |  |
| Baseline | 31.432 | 19.458 | 25.642 | 25.655 | 36.502 | 20.245 | 0.058 | 0.493 | 0.057 | 0.493 |  |
| 3months | 24.865 | 12.641 | 10.557 | 11.681 | 20.674 | 20.205 | 0.017 | 0.016 | 0.313 | 0.016 |  |
| 6months | 18.660 | 11.411 | 4.550 | 3.454 | 10.413 | 8.671 | 0.003 | 0.001 | 0.047 | 0.001 |  |
| 9months | 12.775 | 8.191 | 2.151 | 0.713 | 3.415 | 2.082 | 0.000 | 0.000 | 0.001 | 0.000 |  |
| 12months | 10.817 | 7.900 | 1.478 | 0.472 | 2.235 | 1.582 | 0.000 | 0.001 | 0.002 | 0.001 |  |
| P1==> ATD only (control group) VS CONTROL VS ATD + Azathioprine (1 mg/dl) P2==> ATD only (control group) VS CONTROL VS ATD + Azathioprine (2 mg/dl) P3==> CONTROL VS ATD + Azathioprine (1 mg/dl) VS ATD + Azathioprine (2 mg/dl) | | | | | | | | | | |  |
|  |  |  |  |  |  |  |  |  |  |  |  |

| THYROID VOLUME | ATD only (control group) | | ATD + Azathioprine (1 mg/dl) | | ATD + Azathioprine (2 mg/dl) | |  |
| --- | --- | --- | --- | --- | --- | --- | --- |
| GROUP | Mean | SD | Mean | SD | Mean | SD | Sig. |
| Baseline | 69.43884 | 35.376471 | 51.55512 | 20.193471 | 79.71524 | 47.147563 | 0.260 |
| 12months after remission | 50.23403 | 31.596540 | 41.10229 | 16.514915 | 59.62132 | 31.435700 | 0.460 |
